# Texas Senate Bill 8 significantly reduced travel to abortion clinics in Texas

**DOI:** 10.3389/fgwh.2023.1117724

**Published:** 2023-03-20

**Authors:** Martin S. Andersen, Christopher Marsicano, Mayra Pineda Torres, David Slusky

**Affiliations:** ^1^ Department of Economics, University of North Carolina at Greensboro, Greensboro, NC, United States; ^2^ Department of Educational Studies, Davidson College, Davidson, NC, United States; ^3^ School of Economics, Georgia Institute of Technology, Atlanta, GA, United States; ^4^ Department of Economics, University of Kansas, Lawrence, KS, United States

**Keywords:** abortion, health policy, public policy, digital health, mobility

## Abstract

The *Dobbs v. Jackson* decision by the United States Supreme Court has rescinded the constitutional guarantee of abortion across the United States. As a result, at least 13 states have banned abortion access with unknown effects. Using “Texas” SB8 law that similarly restricted abortions in Texas, we provide insight into how individuals respond to these restrictions using aggregated and anonymized human mobility data. We find that “Texas” SB 8 law reduced mobility near abortion clinics in Texas by people who live in Texas and those who live outside the state. We also find that mobility from Texas to abortion clinics in other states increased, with notable increases in Missouri and Arkansas, two states that subsequently enacted post-Dobbs bans. These results highlight the importance of out-of-state abortion services for women living in highly restrictive states.

## 1. Introduction

**Figure 1:**
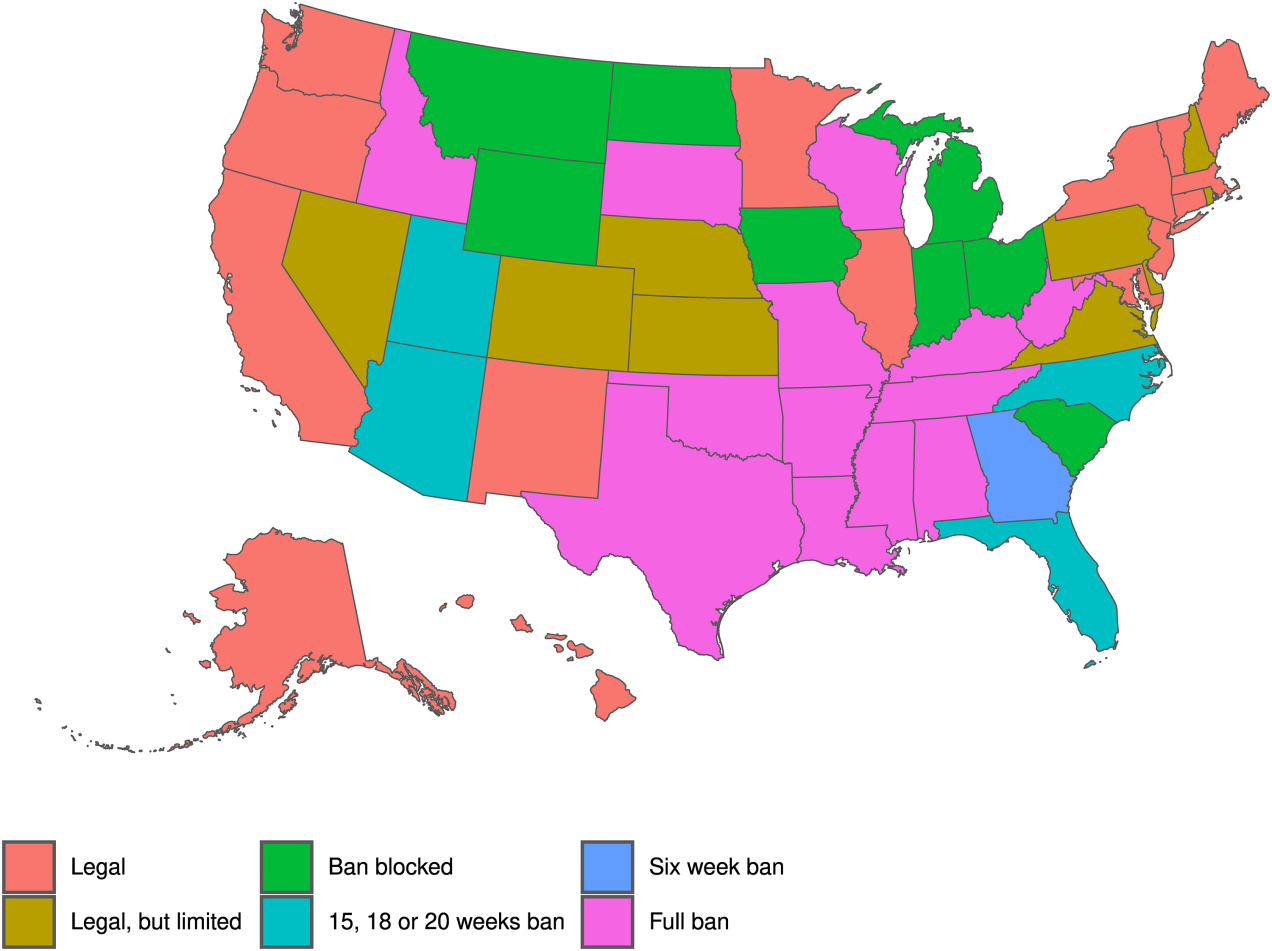
Abortion policies by state, following Dobbs v. Whole Women's Health.

On June 24th, 2022, in the *Dobbs v. Jackson* decision, the United States Supreme Court overturned *Roe v. Wade* and *Planned Parenthood v. Casey*, rescinding the right to abortion (1). Immediately thereafter, trigger laws in 13 states prohibited or severely restricted access to abortion (Figure 1), with elected officials in those and other states considering further restrictions. Although it is too early to see the full effect of the *Dobbs* decision, we can anticipate what is to come by studying an earlier law. In late 2021 the Court allowed Texas’ Senate Bill 8 (SB8) to go into effect, prohibiting abortions in the state after 6 weeks of gestational age. Others have shown (2) that SB8 led to an increase in requests for self-managed medication abortions (3) and travel to abortion providers in four states contiguous to Texas (4). This paper is the first to quantify the impact of SB8 beyond Texas's nearest neighbors. It is also the first to use mobility data to assess the points of origin of patients—both within and outside of Texas.

Abortion access in the U.S. has been a longstanding controversial and divisive issue. In 1973, the U.S. Supreme Court legalized abortion nationwide in the landmark case Roe v. Wade (5). This case established the right to an abortion during the first trimester as protected under a constitutional right to privacy. However, this decision came with criticism, which ultimately reflected in further decisions from the Supreme Court, such as the 1992 decision in the case Planned Parenthood of Southeastern Pa. v. Casey (6). In this case, the Court upheld the legality of abortion throughout the U.S. but changed regulatory standards. Under *Casey*, states could not prohibit women from obtaining an abortion before viability. Still, states did have the right to restrict abortion, as long as a restriction did not represent an undue burden on women seeking abortions. After *Casey*, policies restricting abortion access became more common, particularly in those states where opposition to abortion had been historically strong.

Texas is one of these states. Different abortion restrictions have been implemented across time and, therefore, even before Texas SB8, women faced high barriers to accessing reproductive healthcare. Among the most recent policies implemented in this state are a 2000 parental involvement law, a 2003 two-trip mandatory waiting period, and 1998 and 2009 targeted regulations of abortion providers (TRAP laws). However, its most controversial policy was a 2013 TRAP law, Texas HB2, which required abortion providers to obtain admitting privileges at a hospital located within 30 miles of the abortion facility, among other provisions. As a result, more than half of the abortion facilities closed because the providers could not obtain admitting privileges in nearby hospitals. Then, the distance to the nearest abortion increased for women living in some countries, causing a decrease in abortion rates and increases in birth rates (7–10). In June 2016, the Supreme Court struck down the admitting privileges and distance regulations included in the bill, issuing a majority opinion that the state had failed to demonstrate they served a legitimate interest in regulating women's health and that they imposed an undue burden to access abortion (11). However, even though the policy was struck down, as of June 2018, only three clinics that closed because of Texas HB2 reopened (8).

Although Texas’ abortion landscape has historically been more restrictive than other states, its case study has informed us of the potential impacts that abortion policies in other states could have. For example, Fischer et al. (7) estimated a 1.3 percent increase in births in counties that did not have a provider within 50 miles after H2B implementation. Jones and Pineda-Torres (12) explore the impacts on teenage fertility of targeted regulations of abortion providers (TRAP laws) implemented across the US. H2B is one of the studied policies. Their findings indicate TRAP law implementation increases teen births by 3 percent in TRAP states v. non-TRAP states. Although these studies explore changes in abortion access at different geographic levels, HB2 impacts on fertility are consistent with the impacts of overall TRAP laws.

Besides studies focusing on Texas, an extensive body of work has documented the impacts of abortion policies on abortion access, use, and fertility. For example, in the case of the U.S., different studies have explored changes in these outcomes induced by legalization of abortion in the 1970s (13–18). Furthermore, an array of studies has analyzed the health impacts of abortion access induced by state-level policies such as parental involvement laws [the most recent evidence has been provided by Joyce et al. (19) and Myers and Ladd (20)], and mandatory waiting periods for abortion (21–24). Other studied policies include restrictions on the use of Medicaid for abortion,^1^ gestational limits (25, 26), and compulsory ultrasound requirements (27).

Outside of the U.S., different studies have documented the health and economic impacts of abortion policies in Norway (28), Romania (29, 30), Eastern European countries (31), Spain (32), Mexico (33, 34), and Israel (35). Overall, studies on the U.S. and other countries reach similar conclusions on the causal impacts of abortion policy on abortion access, abortion use, fertility, and economic outcomes.

## 2. Methods

### 2.1. Data

We collected location data on 813 abortion clinics from the restricted version of the Myers Abortion Facility Dataset (36), which included the latitude and longitude of each clinic and information on the services provided by each clinic. We matched these data with weekly mobility data from SafeGraph for locations within 250 meters of an abortion clinic (using the Haversine formula). The SafeGraph data we use do not include personally identifiable information and do not report data on any individual device. To protect the privacy of the individuals in the data, SafeGraph employs differential privacy methods, similar to those used by the Census Bureau, that add noise to the underlying data (37–39). No ethical review was required by the University of North Carolina at Greensboro Institutional Review Board. We discuss other ethical considerations further below.

**Figure 2:**
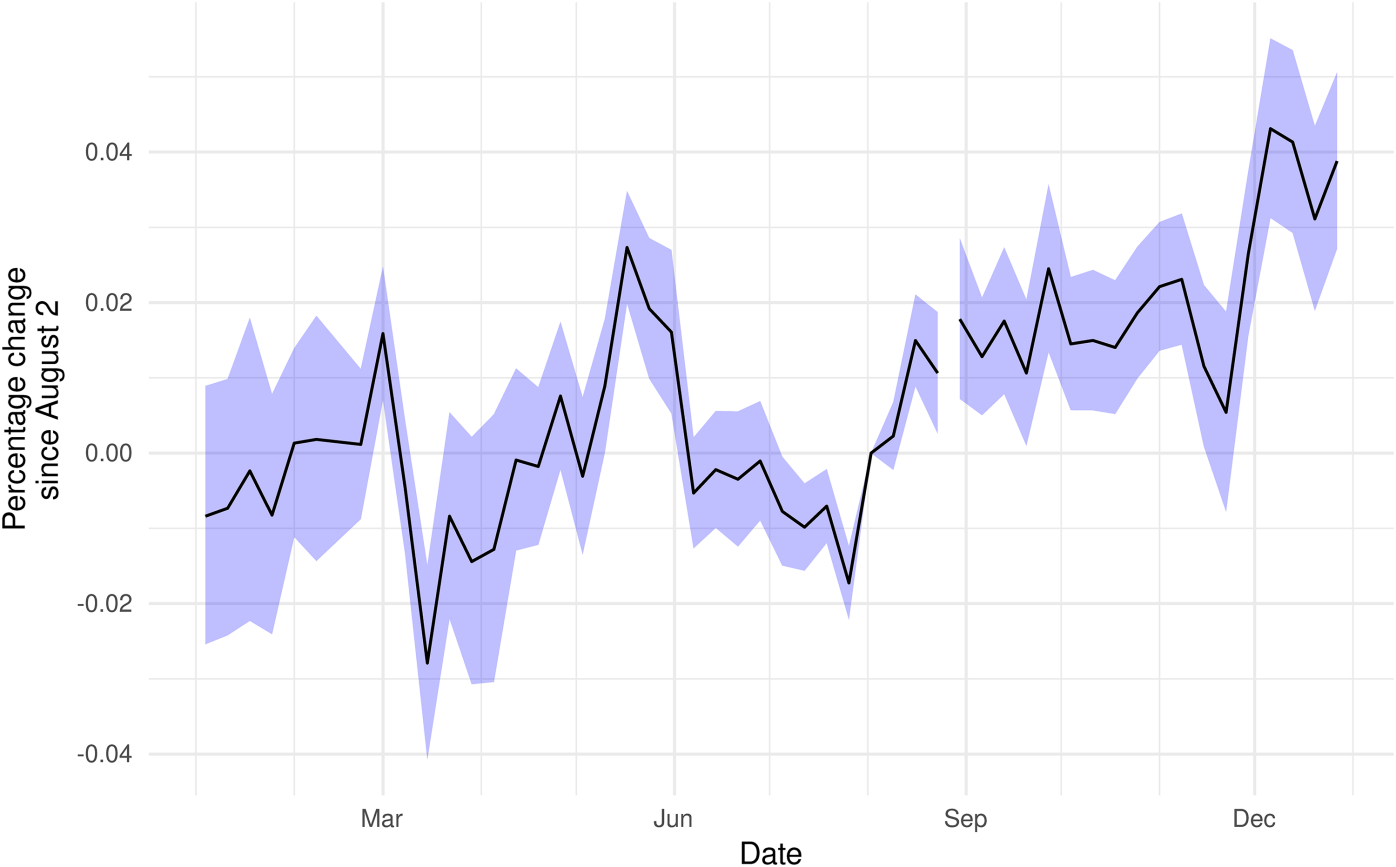
Device counts did not appreciably change following Texas’ SB8. Coefficients are the interaction of an indicator for Texas and date from a two-way fixed effects Poisson regression of total devices seen in each state on date and state fixed effects. Standard errors clustered at the state level.

Our SafeGraph data come from millions of consenting smartphone users using location-enabled apps. These data provide information on the number of unique devices (visitors) that visit each location in the panel on a weekly basis and the total number of visits to each location, which counts returning visitors. Our data do not include individual-level information, nor can it be used to identify individuals. In addition, SafeGraph assigns each device a home location, at the Census Block Group (CBG) level, based on the common nighttime location from the previous 6 weeks (40). SafeGraph reports the number of weekly visitors to each location from home CBGs with more than two visitors traveling to a given location from that CBG. The exact number of users contributing to the SafeGraph panel varies over time therefore, we scale the data to represent the movement of the population in each state by multiplying the counts of visitors to a location by the ratio of state population to the average number of devices observed in the SafeGraph panel in each state and week. We checked for differential changes in the number of devices in the SafeGraph sample over time (Figure 2) and found no evidence of a reduction in devices in the sample in Texas, relative to other states, following the implementation of SB8.

From our initial list of approximately 90,000 locations, we excluded over 70,000 locations with a North American Industry Classification System (NAICS) code starting with “62”, which indicates that the location was a healthcare-related location. We imposed this condition to protect participants in the SafeGraph panel from potential legal liability under Texas’ SB8 law (see “Ethical considerations” below). As a result, our final sample includes weekly mobility data from SafeGraph for 20,334 non-healthcare locations (e.g., restaurants, banks, etc.) within 250 meters of 814 abortion providers in the United States from January 2021 to December 2021 (we omit the week of February 15th, 2021 due to the Texas ice storm). Table 1 reports the number of points of interest by industry in our sample. The majority of locations in our sample come from two sectors–Retail Trade and Accommodation and Food Services–which include retail outlets like Target and Walmart, coffee shops like Starbucks and Peets Coffee, and restaurants including McDonald's, Red Robin, and Applebee's. For our analysis, we aggregated our data to the nearest clinic level so that our final dataset is a panel dataset of mobility in proximity to abortion clinics over time.

**Table 1:** Industry distribution of the safe graph sample.

Industry sector	# POIs	%
Utilities	2	<0.01
Construction	245	1.06
Manufacturing	581	2.51
Wholesale Trade	167	0.72
Retail Trade	6,101	26.30
Transportation and Warehousing	283	1.22
Information	397	1.71
Finance and Insurance	1,456	6.28
Real Estate and Rental and Leasing	783	3.38
Professional, Scientific, and Technical Services	601	2.59
Management of Companies and Enterprises	50	0.22
Administrative and Support and Waste Management and Remediation Services	95	0.41
Educational Services	774	3.34
Health Care and Social Assistance	0	0.00
Arts, Entertainment, and Recreation	1,257	5.42
Accommodation and Food Services	6,236	26.90
Other Services (except Public Administration)	3,597	15.50
Public Administration	351	1.51

# POIs is the unduplicated number of points of interest in our sample.

We developed a novel approach to study visitors’ origin points and destinations to abortion clinics in the Myers Abortion Facility dataset. Not only does the approach allow us to understand any decline in the number of devices (and thus individuals) at abortion clinics due to SB8, but also the alternative destinations selected by those device users. In short, we know where would-be abortion clinic visitors go when they can no longer visit a clinic in Texas. To our knowledge, no other study to date has taken this approach.

### 2.2. Ethical considerations

The use of data derived from health information technologies (HIT) to study abortion access has been of great concern to regulators, policymakers, and the public (41–43). These concerns became particularly acute following press reports about data sharing by period tracking apps (44) and the use of Facebook messages in a recent abortion prosecution (45). These concerns led some geolocation data providers to restrict data collection around sensitive locations (43, 46).

We adopted a research design that reduces potential risks to women seeking access to abortion. First, using cellphone-based measurements, rather than clinic-level data, protects women seeking abortions because we do not know the reason for a visit to a clinic—women may be near an abortion clinic to visit a nearby Starbucks or visiting the clinic itself for other healthcare services. A cellphone-based approach also reduces the administrative burden on clinics and allows us to collect data on a broader range of locations than would be feasible, collecting data from each clinic individually, thus providing a landscape of changes in mobility.

Second, we use locations near abortion providers as a proxy for mobility to abortion clinics because SafeGraph no longer provides mobility data to family planning centers (which include abortion clinics). We exclude healthcare-related locations in case there is misclassification of some abortion providers. Our assumption is that changes in the number of devices visiting locations near an abortion clinic will be proportional to changes in the number of devices visiting a clinic (47), which is plausible since some of the nearby locations include coffee shops and other locations where people may loiter before or after visiting a clinic. Because we cannot deduplicate the count of visitors near an abortion clinic, we cannot directly convert our mobility estimates into anticipated changes in abortions as a result of Texas’ SB8 law.^2^

Our approach, which allows us to understand the potential mobility patterns surrounding the adoption of SB8, also prohibits us from identifying individuals and exact clinic visit patterns. However, it is precise enough to estimate the effect of SB8 without being so precise as to be useful for law enforcement or those who would seek to use the data to target oft-traveled clinics for anti-abortion protests.

We view geolocation data as a new and valuable data point for health services and public health research. These data have been frequently relied on by the medical and health policy communities during the COVID-19 pandemic, demonstrating their utility for medical researchers (49, 50). These data also have potential future applications in assessing access to care using observed, rather than hypothesized, movement patterns.

### 2.3. Descriptive statistics on abortion in the United States

The abortion rate in the U.S., i.e., abortions per 1,000 15–44-year-old women, has sustained a decreasing trend since the 1990s. The average abortion rate between 2000 and 2019 was 12 abortions per 1,000 women of reproductive age.^3^ The lowest abortion rate was observed in 2019, with 9.8 abortions per 1,000 women of reproductive age. Abortion rates in Texas were consistently higher than the national rates up to 2013. However, starting in 2014, abortion rates in Texas have been below the national rates. For instance, between 2000 and 2013, the average abortion rate in Texas was 14.9 compared to a national average rate of 12.9 abortions per 1,000 15–44-year-old women. However, from 2014 to 2019, the average abortion rate was 9, compared to a national average rate of 9.9 abortions per 1000 15–19-year-old women. This decrease in abortion rates in Texas since 2013 is likely associated with the abortion policies implemented in Texas in the last decade, particularly Texas H2B, as described in section 1.

### 2.4. Empirical methods

We assessed the effect of SB8 on visits near abortion clinics in a difference-in-differences framework (23), which identifies the causal effect of SB8 based on differences in changes in movement patterns to clinics in Texas, compared to other states, while controlling for aggregate time effects. Our implementation uses differences between clinics in Texas and other states before and after August 30th, 2021 (the beginning of the week containing September 1st, 2021, when SB8 took effect). Our identifying assumption is that in the absence of SB8, movement patterns near abortion clinics would be the same in Texas and other states. While this assumption is not directly testable, we can test for differences over time before the implementation of SB8.

Our difference-in-differences regression specification is:

$$E[Y_{it}|Post_{t},TX_{i},\delta_{i},\gamma_{t}]=\exp(\beta_{1}Post_{t}\times TX_{i}+\delta_{i}+\gamma_{t})$$

Where $Y_{it}$ is the number of visitors or visits to location *i* in week *t*, $Post_{t}$ is a dummy for the post-period, $TX_{i}$ indicates if location *i* is in Texas, $\delta_{i}$ is a set of unit fixed effects (which control for time-invariant differences across units), $\gamma_{t}$ is a set of week fixed effects (which control for time-varying differences across units). The coefficient of interest, $\beta_{1}$, identifies the proportional change in visits to abortion clinics in Texas, relative to other states, after SB8, compared to trends before SB8 took effect.

The difference-in-differences model identifies the causal effect of treatment on the treated under a parallel trends assumption—essentially, trends in “control” units are parallel to trends in “treated” units in the counterfactual scenario when treated units are not actually treated. While we cannot directly test this assumption, we can look at “event studies” that plot the evolution of an outcome variable in treated and control units over time. If the pre-trends are parallel, then it is more likely that the post-treatment trends in the counterfactual scenario are also parallel. For our event studies, we used the same specification as above, but replace $Post_{t}$ with $\gamma_{t}$, and $\beta_{1}$ becomes a vector of differences in outcomes for treated versus control units. We normalize the week of August 3rd, 2021, to be zero so that the unit fixed effects are identified. We rely on a Poisson estimation which assumes a discrete probability distribution of the probability of an event occurring during a fixed time interval, such as the number of visits/visitors to a location in a week. We clustered the standard errors at the state level.

To examine the extent to which people traveled to other states following the SB8 decision, we also estimate models of the form:

$$\begin{array}{rl} & E[Y_{ijt}|Post_{t},TX_{i},\delta_{i},\gamma_{t}]=\\ & \;\exp\;(\beta_{1}Post_{t}\times TX_{i}\times TX_{j}+\beta_{2}Post_{t}\times (1-TX_{i})\times TX_{j})\\ & \;+\beta_{3}Post_{t}\times TX_{i}\times (1-TX_{j})+\delta_{ij}+\gamma_{t} \end{array}$$

Where $Y_{ijt}$ is the number of visitors from home *j* to location *i* in week *t*, $Post_{t}$ is a dummy for the post period, $TX_{j}$ is an indicator that the home location was in Texas, $\delta_{i}$ is a set of unit fixed effects, and $\gamma_{t}$ is a set of week fixed effects. The coefficients $\beta_{1}$, $\beta_{2}$, and $\beta_{3}$ correspond to the change in visitors to Texas locations from Texas devices, non-Texas locations from Texas devices, and Texas locations from non-Texas devices. Visitors to non-Texas locations from non-Texas devices are the excluded reference group. As in the previous specification, we rely on a Poisson estimation and use two-way clustering at the source and destination state levels to compute our standard errors. These methods assume that in the absence of Texas’ SB8, the trend in the number of visits/visitors to a location would have been the same in locations in Texas as what is observed in locations in other states.

Our final analysis, which provides insight into the impact of the *Dobbs* decision on women in Texas, generalizes the previous model by estimating changes in mobility near abortion clinics in each state for Texas versus non-Texas residents. This method of analysis, to our knowledge, is the first of its kind to assess both the point of origin and potential destinations of those seeking abortion care across state lines.

## 3. Results

**Figure 3:**
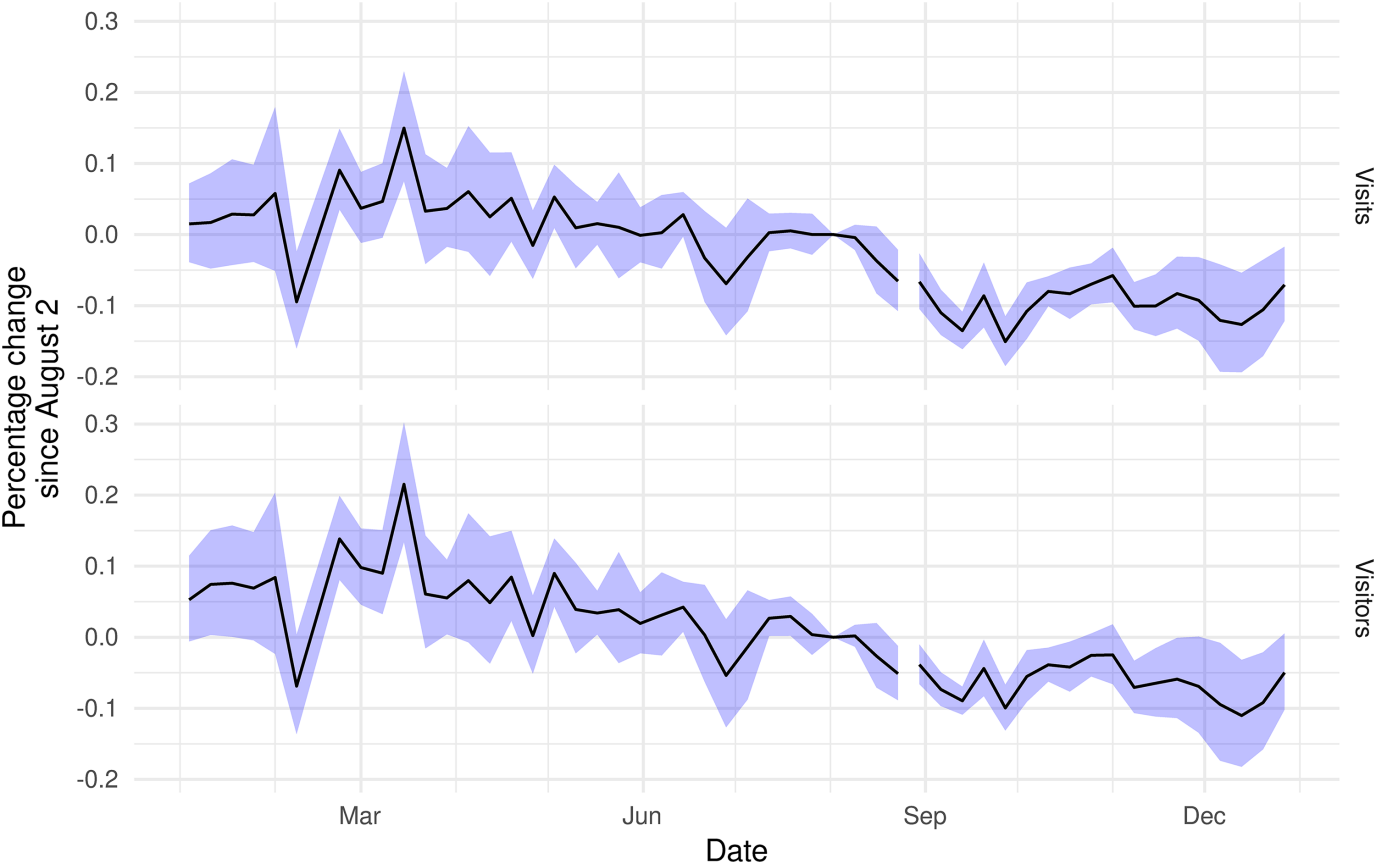
Visits (total devices) and visitors (unique devices) to areas near abortion clinics in Texas, relative to clinics in other states and to August 2nd, 2021. Coefficient estimates for the interaction of date with an indicator for Texas from two-way fixed effects Poisson regression, including clinic and date controls. Standard errors are clustered at state level.

Figure 3 plots the relative change in the average number of visits (top) and visitors (bottom) near abortion clinics, by week, in Texas versus all other states. For both outcomes, there was a visually apparent decline beginning in early August of 2021 that was sustained throughout the Fall of 2021. The additional dip in early December corresponds to the Supreme Court oral arguments in *Dobbs v. Whole Women's Health,* the case that led to the overturning of both *Roe v. Wade* and *Planned Parenthood v. Casey*. While there are occasional statistically significant differences from zero in the pre-period, these events are concentrated in February and March of 2021.

**Figure 4:**
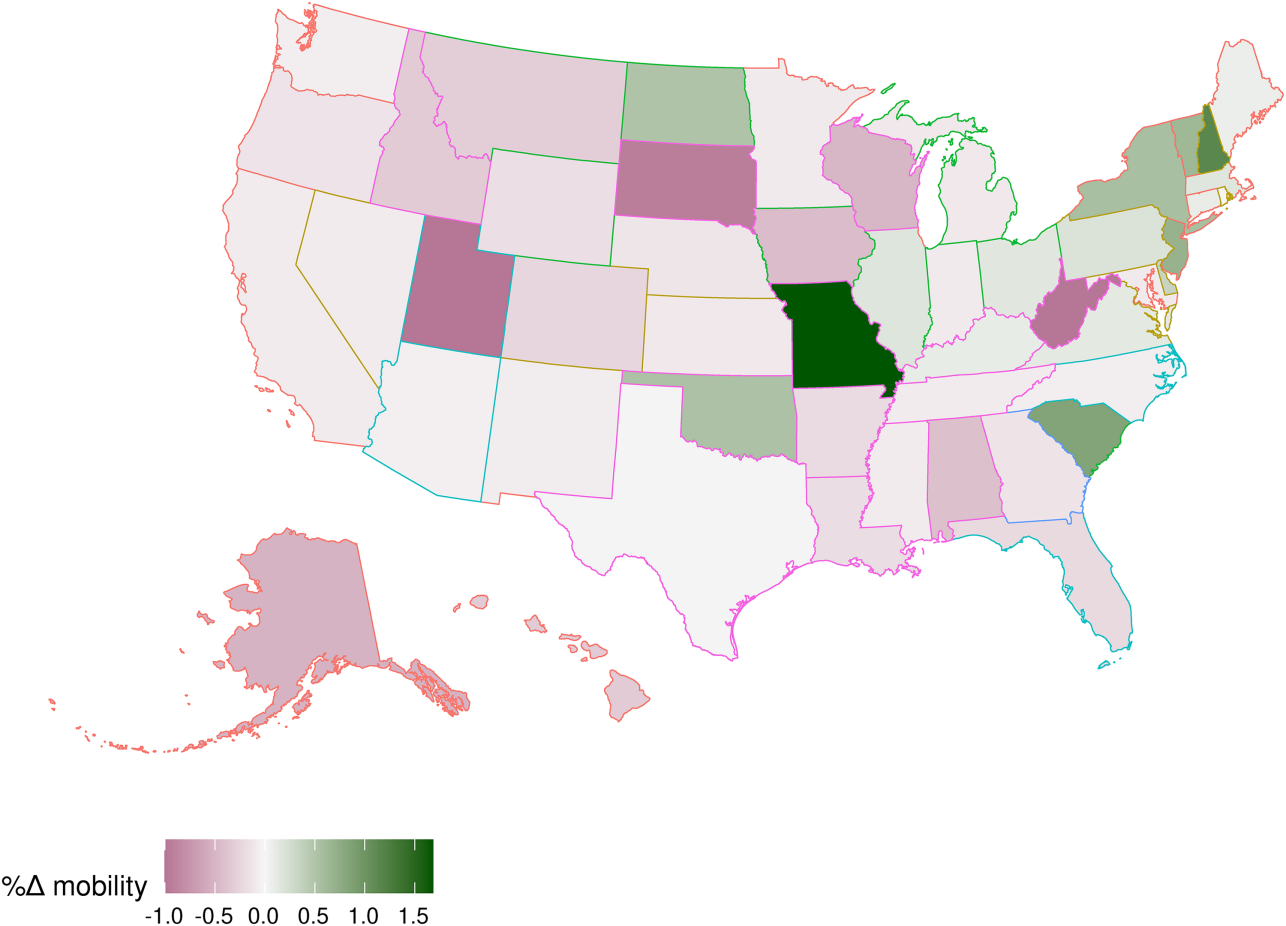
Percentage change in mobility near abortion clinics by state for Texas devices relative to non-Texas devices (outline colors correspond to Figure 1). Each state is shaded according to the predicted percentage change in mobility from Texas to the state following SB8's implementation. Estimates are from destination-state specific two-way fixed effects Poisson regressions of visitors on a dummy for a Texas origin interacted with Post SB8, including (destination) clinic-by-origin Census block group and date fixed effects.

Table 2 presents difference-in-differences estimates of the effect of Texas’ SB 8 law on visits near abortion clinics. The first two columns demonstrate that SB8 led to a 10–11 percent reduction in mobility near abortion clinics. The final column demonstrates that there was a substantial reduction in visitors near abortion clinics in Texas for devices typically used in Texas (9.1 percent, 95% CI: 3.4–14.7, *p* = 0.003) and outside of Texas (10.8 percent, 95% CI: 5.7–15.8, *p* < 0.001). At the same time, Texas residents significantly increased visits near abortion clinics outside of Texas (6.9 percent, 95% CI: 3.0–10.9, *p* < 0.001). Figure 4 builds on the third column's result and demonstrates substantial increases in mobility to several states, notably Missouri, but also several Northeastern states, Oklahoma, and South Carolina. Among these states, Missouri and Oklahoma had post-*Roe* trigger ban laws and, as of September 2021, have banned or substantially reduced access to abortion in those states. Our novel mobility-data-focused method, therefore, shows that destinations that may have provided abortions to Texans in the wake of SB8 are no longer options after the *Dobbs* ruling and resulting trigger laws.

**Table 2:** Difference-in-differences estimates.

	(1)	(2)	(3)
	Visits	Visitors	Visitors by origin
Visits to Texas clinics	−0.108 (0.028)	−0.099 (0.030)	
Texas residents visiting Texas clinics			−0.091 (0.029)
Texas residents visiting clinics outside Texas			0.069 (0.020)
Non-Texas residents visiting Texas clinics			−0.108 (0.026)
# of observations	41,463	41,463	51,229,459
# of clinics	813	813	813
# of home locations (census block groups)	–	–	189,111

All coefficients are interacted with an indicator for after Texas SB8 took effect. Models for columns (1) and (2) included clinic and week fixed effects. The model in column (3) included origin census block group by clinic and week fixed effects. Standard errors clustered at the state (columns 1 and 2) or origin and destination state (column 3) level in parentheses.

## 4. Discussion

Following the *Dobbs* decision, abortion bans are becoming more common. Therefore, it is important to understand the consequences of restrictions on abortion access, with special attention paid to the availability of alternative means for women to access abortion services through out-of-state travel. This study—to our knowledge, the first of its kind to assess potential cross-state clinic visit effects with mobility data—demonstrates a significant reduction in visits to areas around abortion clinics in Texas following the implementation of SB8. However, the reduction in access to care within the state was partially offset by increases in mobility to clinics outside of Texas. Notably, we observed increases in mobility to states such as Missouri, South Carolina, and New York, which do not border Texas and have not been included in previous studies of changes in abortion visit patterns due to SB8 (4). As these results reveal, women living in restrictive states have historically relied on out-of-state abortions abortion services. However, at least two of those destinations–Missouri and Oklahoma–are no longer an option due to post-*Dobbs* trigger laws, while the situation in Kansas has temporarily stabilized with the defeat of a constitutional amendment that would repeal abortion protections in the state (52). Therefore, as more states implement abortion bans, the abortion landscape will continue turning more restrictive, limiting out-of-state options for residents of such states.

Our paper is also one of the first to demonstrate the utility of geolocation data for monitoring access to healthcare services. Measuring access to ambulatory healthcare services is challenging since people have various insurance arrangements—including no insurance at all—and states do not engage in centralized data collection for ambulatory services. Geolocation data provides from a diverse set of devices and can measure movement both to ambulatory service providers, subject to privacy concerns, and close to those providers. For this reason, geolocation data should be considered in all future studies of access to healthcare. However, these efforts must be tempered by an appreciation of privacy concerns. In our implementation, for example, we do not directly measure access to abortion care but rather infer it based on how movement patterns in the vicinity of an abortion clinic changed following SB8. This approach may be useful in future research on access to abortion in both the United States and other countries.

Our analytic strategy has some limitations since we cannot, by design, identify individuals visiting abortion clinics, and our definition of near encompasses people who may be visiting a clinic, protesting at the clinic, and visiting other locations. First, our estimates of mobility near clinics may be biased if SB8 resulted in a reduction in abortion protestors near clinics in Texas or led to an increase in abortion protestors from Texas traveling to clinics in other states. Second, our results do not imply that there will be a commensurate reduction in abortions in Texas since we do not demonstrate that our mobility data are correlated with abortions by state. Third, because of privacy protections, our measure of out-of-state movement is likely to be understated because SafeGraph does not report links with two or fewer devices after differential privacy has been applied. Fourth, women seeking abortions, particularly in the aftermath of SB8, may have disabled location services, leading us to underestimate movement to clinics outside of Texas following SB8.

## Data Availability

The data analyzed in this study is subject to the following licenses/restrictions: The datasets are available with a subscription to Dewey Data. Requests to access these datasets should be directed to www.deweydata.io.

## Footnotes

^1^See section 2.1 in Jones and Pineda-Torres (12) for a detailed list on the studies of these policies.

^2^See Andersen et al. (48) for a paper that converted mobility changes into abortion counts.

^3^Own calculations using abortion counts on abortion occurrences from the CDC Abortion Surveillance System (51) and population data from SEER program.
